# *Procedural Justice and Prison Legitimacy: Towards a Democratic Model of Inmate Participation* ^†^

**DOI:** 10.1093/ojls/gqaf013

**Published:** 2025-05-23

**Authors:** Chloé Deambrogio

**Keywords:** penal legitimacy, compliance, procedural fairness, prison democracy, Global South

## Abstract

The procedural account of prison legitimacy proposes that inmates’ compliance with correctional institutions depends more on whether they feel that prison guards treat them fairly during their daily interactions than on whether the guards’ decisions are ultimately favourable to them. In *Crime, Justice, and Social Order*, Anthony Bottoms and Alison Liebling provide a compelling overview of their work in this area, highlighting the importance of respectful relationships for building feelings of trust in penal institutions and advancing a humanitarian account of legitimacy that is sensitive to the moral and relational dimensions of order maintenance. Despite their important contribution, Bottoms and Liebling’s procedural approach advances a precarious notion of legitimacy that depends too heavily on the fair treatment of inmates by prison guards and too little on methods of inmate participation that might help the institution align its values with those of prisoners, creating a more stable, and truly normative, commitment towards compliance.

## 1. Introduction

*Crime, Justice, and Social Order. Essays in Honour of AE Bottoms* is a collection of essays edited by Alison Liebling, Joanna Shapland, Richard Sparks and Justice Tankebe, published by Oxford University Press. The book honours the life and work of Professor Sir Anthony Bottoms, a leading British criminologist and ‘citizen scholar’ who has contributed with his outstanding research to the advancement of criminological knowledge, while maintaining an active public engagement throughout his lifelong career. The collection includes 14 essays spanning a wide range of topics, including theoretical pieces focusing on Bottoms’s work in the area of moral philosophy and political theory, and empirical papers building on his research on policing, prison social order and desistance from crime. The essays are written by authors who have either worked directly with Bottoms or had the opportunity to engage with topics that do justice to Bottoms’s research interests. They all ‘pay tribute’ to aspects of Bottoms’s work and celebrate the accomplishments, intellectual contributions and humanity of this well-loved scholar and, for many of the authors, close friend. Given the breadth of scholarship included in the collection, in this review article I will concentrate on the sections of the book that build on Bottoms’s research on legitimacy, compliance and prison social order, and on the literature that inspired and/or stemmed from them. Specifically, I will concentrate on the theory of legitimacy developed by Alison Liebling in her essay ‘Penal Legitimacy, Well-being and Trust: The Role of Empirical Research in “Morally Serious” Work’,^1^ drawing from other chapters in the collection when relevant for my argument.^2^ As we will see, Liebling’s research has largely confirmed, and in many ways expanded, the theory of legitimacy developed by Richard Sparks, Anthony Bottoms and Will Hay in their book *Prisons and the Problem of Order*, an ethnographic study of two English maximum security prisons conducted in the late 1980s.^3^ According to these authors, in English prisons legitimacy and compliance depend primarily on the quality of the relationships established between correctional staff and inmates, and specifically on whether inmates feel that police officers and prison guards treat them with fairness, humanity and respect. This finding advances a procedural approach to legitimacy and compliance that places a premium on humanising social interactions to reduce frictions and conflicts that might lead to consent withdrawal and social disorders.^4^

As I argue in this review article, on the one hand, this approach to legitimacy is helpful because it strives to create safe prison societies where staff treat inmates with humanity and inmates abide willingly to the rules of the institution. On the other hand, a theory of social order that places a premium on procedural fairness is problematic because: (i) it advances a precarious notion of legitimacy that depends too heavily on the behaviour and professionalism of staff; and (ii) it induces compliance by manipulating subordinated subjects into accepting the decisions of the prison administration rather than by stimulating a truly normative commitment to the rules of the institution.

I propose that to obtain more stable versions of legitimacy and compliance we need to advance a democratic model of prison governance that, departing from the managerial system of vertical power relations that dominates prison systems in the Global North, engages inmates in the daily running of the institution by giving them the opportunity to shape the rules that should guide prison life. To advance this claim, I draw from two comparative case studies from the Global South, specifically Ethiopia and Brazil, that demonstrate that, even in conditions of austerity, it is possible to involve inmates in the governance and management of the prison in ways that enhance, rather than sacrifice, safety and social order.

## 2. Legitimacy and Procedural Fairness in English Prisons

Prisons in England and Wales are in a critical state. Recent reports show a prison system characterised by a growing prison population, chronic overcrowding, staff shortages, high levels of self-harm and record rates of violence among inmates.^5^ Officials describe prisons as frightening and depressing places, where many inmates spend between 20 and 22 hours a day locked up in their cells, with little or no opportunities for education or training.^6^ Prominent commentators have expressed concerns about this state of affairs, warning that there is a serious risk that, if triggered, prisoners’ frustrations might escalate to violent riots, and that the correctional staff might be unable to contain the rebellion due to drastic reductions in the prison workforce.^7^ According to former Chief Inspector of Prisons Nick Hardwick, ‘we are very close to the conditions prior to the 1990 riots in Strangeways. It’s a dangerous time. You’ve got a general level of frustration, so if you get a spark, that’s when stuff will kick off.’^8^ English prisons, this statement suggests, are facing a deep legitimation crisis that might prompt inmates to retrieve their consent and engage in various forms of civil and uncivil disobedience.

It was in a similar climate that Sparks, Bottoms and Hay wrote *Prisons and the Problem of Order*, a widely influential book that sought to examine the relationship between legitimacy and social order and the normative basis of compliance in English prisons. In the 1980s, the decade preceding the publication of the book, the English prison system experienced a decline in the confidence in its administrative abilities that prompted the eruption of a series of disorders, including riots and protests, in all but five of the country’s high-security dispersal prisons.^9^ These revolts brought concerns about control and security to the forefront of the prison management agenda, prompting the Home Office to commission a formal inquiry aimed at identifying the root causes of the disturbances. The inquiry, known as the Woolf Report, found that prisoners’ protests stemmed primarily from a diffuse sense of frustration with the conditions of their confinement, which was characterised by ‘chronic overcrowding, unsanitary conditions, and indecent treatment by staff’.^10^ The way staff treated prisoners, and particularly the lack of respect that permeated their daily interactions with prison guards, were particularly important factors in prompting the unrest.^11^

Like the Woolf Report, *Prisons and the Problem of Order* was commissioned to explore the reasons for the prison disturbances, and to identify which forms of order, if any, could be said to be ‘more legitimate, and sustainable, than others’.^12^ The research was conducted in two dispersal prisons that presented dramatically different characteristics: Albany, an institution with a history of social disorders and a restrictive regime of control, and Long Lartin, a ‘liberal’ prison that had not experienced any major disorders and that saw ‘choice, responsibility and self-respect’ as central values to be advanced in the daily running of the institution.^13^ By comparing these ‘polar extremes’, the authors were able to identify the key factors that distinguished prisons that experienced major disturbances from those that had remained relatively stable.^14^ They found that the relative stability of Long Lartin could be explained by its perceived legitimacy amongst prisoners, which in turn could be explained by the relative fairness with which prisoners felt they were treated by staff in their daily social interactions.^15^ It was only by fostering legitimate social relations that prisons could generate *normative commitments towards compliance*. Legitimacy, the authors argued, was a precondition for stable social order in prisons.^16^

In her essay, ‘Penal Legitimacy, Well-being and Trust’, Liebling examines how the theoretical insights developed by Sparks, Bottoms and Hay in *Prisons and the Problem of Order* have been empirically tested and expanded in recent research. In particular, she discusses an evaluation study she conducted (with Arnold’s assistance) on a system of incentives and earned privileges (IEP) introduced in five prisons in 1995–6.^17^ Largely confirming Sparks, Bottoms and Hay’s findings, the study found that rather than leading to improved behaviour, the IEP programme increased perceptions of unfairness amongst prisoners who, as a result, started rejecting the rules of the institution.^18^ Prisoners’ dissatisfaction with the IEP programme was particularly pronounced in the wings where the relationship between staff and prisoners was most strained, with officers locking themselves in their offices and using formal methods of prison discipline rather than open communication and persuasion.^19^ This factor illustrated the centrality of the quality of social relations for prisoners’ perceptions of institutional legitimacy. According to Liebling’s observations,

prisoners felt treated fairly when staff treated them respectfully and courteously; when they knew them as individuals, gave reasons for decisions, and rationally exercised ‘mercy’, or used their discretion intelligently. *Formal power operated through informal means: via decent relationships*. When staff withdrew into offices, resorted to formal methods of discipline, and then demanded compliance with new and unwelcome policies, prisoners withdrew their consent.^20^

The key moral dimensions identified in the study—fairness, respect and social order—were related in ways that confirmed the legitimacy thesis developed by Sparks, Bottoms and Hay, namely ‘that fairness, relationships, and respect are related to compliance with regimes’.^21^ Fair and respectful interactions build trust and trust, Liebling argues, ‘is fundamental to legitimate prison functioning … it acts as a lubricant, smoothing the flow of power, encouraging social cooperation, and making intelligent distinctions possible’.^22^

As this discussion shows, the notions of legitimacy and compliance emerging from both Sparks, Bottoms and Hay’s and Liebling’s research depend heavily on fair treatment by prison guards in their daily encounters with prisoners. This finding is helpful because it identifies clear areas of intervention that can facilitate the building of consensus through fair procedures and respectful social interactions, rather than through coercive measures. However, the notion that legitimacy and compliance can be induced through positive staff–inmate relationships presents three fundamental problems in need for consideration. First, it advances a procedural approach to legitimacy that is vulnerable to break down should an encounter between officers and prisoners generate conflicts and disagreements. According to American social psychologist Tom Tyler, one of the main findings of procedural justice research is that

every encounter that the public have with the police [or other legal authorities] … should be treated as a socialising experience that builds or undermines legitimacy. Each contact is a ‘teachable moment’ in which people learn about the law and legal authorities.^23^

As argued by Bottoms and Tankebe, this finding is problematic because it

places an awesome responsibility on the police and other criminal justice services. Why? Because its message is that any action by any employee of a criminal justice organization could potentially contribute (to a greater or lesser degree) to either the enhancement or the diminution of the perceived legitimacy of the service as a whole, in the eyes of a relevant audience. Indeed, in the worst-case scenario, *a single incident could severely damage the legitimacy of a whole organization*.^24^

Relying heavily on the presence of smooth social interactions and a professional workforce for the maintenance of social order presents significant risks, especially in a context, such as the English prison system, where reductions in the number of correctional officers has put exceptional pressures on an already strained workforce.^25^ The result is a precarious system that depends excessively on the ‘good behaviour’ of exhausted, and often inexperienced, prison guards for its legitimacy and survival.

Second, a model of social order based on smooth interactions between police officers and prisoners is unlikely to induce a truly normative commitment towards compliance. As argued by Ian O’Donnell in his book *Prison Life*, to ensure that compliance is truly normative, prisoners must comply with authorities because they share the overall values, beliefs and priorities of the institution.^26^ Applied to the prison context, normative compliance means that

prisoners believe that the rules by which they live are issued by a legitimate authority with which they identify. They fall in line willingly, allowing individual priorities to be trampled by the collective. They comply because they believe this to be the correct thing to do rather than out of fear, the prospect of a reward, or thoughtless habit.^27^

Although respectful and fair treatment are likely to induce prisoners to accept the authorities’ decisions, they are less suited to encouraging a deep and long-lasting commitment that reflects shared values and beliefs with the prison administration. This is because while in this model legitimacy is based on dialogue and relationships between staff and prisoners, it is not based on the kind of communication and exchange that is likely to align the priorities of the institution with those of the individuals kept in its captivity. Even though Sparks and Bottoms recognise that ‘a defensible and legitimate prison regime demands a *dialogue* in which *prisoners’ voices* … are registered and have a chance of being responded to’, their accounts tend to focus on inmates’ feeling of ‘being heard’ rather than on their effective power to stimulate change.^28^ As the next sections will show, when prisoners’ ability to influence managerial decisions is limited, their compliance with the institution is more likely to stem from manipulation, lack of alternatives and limited expectations than from a normative commitment based on shared values and beliefs.

### A. Manipulation

The procedural justice literature has repeatedly demonstrated that people have a psychological tendency to accept unjust decisions if the process through which such decisions were reached is perceived to be fair.^29^ Hence, by introducing procedural changes that give an impression of justice and fairness, authorities can manipulate subjects into accepting decisions that go against their long-term interests. As argued by Ronald Cohen,

giving people the impression that a decision will be affected by their expressed opinions, or that their opinions will be seriously considered, or both, provides people with illusions of control. It also seems to provide them with ‘illusions of dignity’. People are led to believe not only that their actions will have an impact on a decision but also that they are receiving the dignity due a responsible adult citizen, an important part of which involves respect for and serious consideration of one’s opinions.^30^

In this view, procedural fairness is pursued not because of its ability to promote the moral values of justice, equality and respect, but because of its effectiveness in advancing managerial concerns of institutional control. Procedural fairness has become a strategic instrument in the hands of power holders, who use it to legitimise unfair decisions in the eyes of subordinated subjects.

Prison managers in England and Wales often use performative rituals that give ‘illusions of dignity’ and respect to manipulate inmates into compliance. One way in which this is done is by stressing the importance of civility and good manners for the development of respectful relationships between staff and inmates, while strategically deploying them as an instrument of social control aimed at inducing the prison population into accepting the decisions of the administration.^31^ As highlighted by Gabrielle Watson in *Respect and Criminal Justice*, although prisons’ emphasis on civil treatment signals a humanitarian concern on the part of the system to advance moral values such as dignity and respect, a closer look at official discourse suggests that the language of respect has become a rhetorical device used to pursue instrumental objectives of order maintenance.^32^

The definition of respect provided by Joe Pilling, former Director General of the English Prison Service, clearly illustrates this tendency. According to Pilling, prison guards should cultivate respect by addressing inmates ‘courteously, by the name they prefer, [by] asking not ordering; [by] listening to what they are saying; [by] being sensitive to their feelings about being locked up’. This courteous treatment, Pilling argues, ‘is the key to *healthy relationships* … the key to *good control*’.^33^ Pilling’s association of respectful relationships with ‘good control’ suggests that rather than stemming from a genuine desire to enhance the moral quality of police–inmate interactions, his (and other prison administrators’) emphasis on civil treatment stems from a managerial concern to induce compliance in the prison population and maintain social order. This approach is manipulative in the sense that it uses ‘politeness’ to avoid resistance and rebellion, rather than seeking strategies that might align the institution’s priorities with prisoners’ needs and values.

### B. Lack of Alternatives and Limited Expectations

Another reason why prisoners might comply with a system that places a premium on civil staff–inmate relationships (while ignoring fundamental needs such as education and training) is related to prisoners’ lack of alternative options. If all prisoners have ever experienced is a system of vertical power relations driven by a managerial ethos, they are likely to be satisfied with the most humane version of this model, even though alternative models could lead to more empowering social arrangements. In other words, the fact that prisoners value primarily how the correctional staff treat them shows that their needs, hopes and expectations are limited by the model of governance under which prisons operate and by what prisoners perceive as being within reach in their present environment.

This leads us to a third and related explanation for prisoners’ responses: limited expectations. If expectations are low, being treated fairly and civilly in a safe environment is considered to be enough: legitimacy flows from the normalisation and routinisation of living under unjust social conditions.

The tendency to passively accept the rules of the regime becomes particularly pronounced when prisoners’ limited expectations combine with a sense of powerlessness and fear. Sparks and Bottoms provide a compelling example of this dynamic in their discussion of the Vulnerable Prisoner Unit (VPU) at Albany, a segment reserved for long-term prisoners who have been convicted for serious offences and need to be isolated from the rest of the inmate population for their own protection.^34^ Due to their ‘powerless state’, when VPU prisoners see the implementation of even more restrictive regimes,

they *accept[…] the inevitable*, knowing that any protest could lead to their being transferred to another prison, where conditions for them could easily be worse. ‘Dull compulsion’ [is] therefore absolutely the main reason for the passive response of these prisoners who [feel] they *[have] no choice* but to put up with their fate.^35^

In the VPU, inmates’ compliance with the rules of the institution stems from their lack of alternatives, limited expectations and powerlessness, not from genuine beliefs in the legitimacy of the regime.

The notion that inmates’ compliance might stem from a lack of alternatives and limited expectations explains why prison systems often experience the most dramatic legitimacy crises when newly introduced privileges are taken away: once prisoners have seen what could be, they are less likely to accept what has always been.

This idea finds support in a series of interviews conducted by Bethany Schmidt with prisoners who were involved in a democratic experiment conducted by the non-profit organisation User Voice in three English prisons.^36^ Interestingly, when asked ‘what kind of penal change would be necessary to address the legitimacy deficit as it currently exists’, the prisoners did not describe a procedurally fair system in which they were treated with civility and respect, and/or a managerial regime that guaranteed minimum levels of safety and protection from other inmates.^37^ Rather, they spoke about a ‘model of prison governance and social order that was inclusive, participatory, mutually accountable, civically minded, and democratic’.^38^ This shows that when exposed to the benefits of democratic participation, prisoners start asking for more than personal safety, civil treatment and professional competence: they want to have a voice in shaping their own destiny. When expectations are raised, even the standards for legitimacy and normative compliance are raised.

## 3. Reforming Prison Governance: Towards a Participatory Model of Prison Democracy

A third and final issue with Sparks, Bottoms and Hay’s and Liebling’s research is that, by basing reform proposals on prisoners’ perceptions of their confinement, it naturally limits the agenda to what is available in the current environment. As we have seen, one of Liebling’s central claims is that by smoothing staff–prisoner relationships we can enhance the moral quality of prisons. This claim is based on the notion that by identifying what prisoners perceive as valuable we can develop policies that will maximise the quality of prison life and enhance feelings of legitimacy in the institution.^39^ While prisoners’ voices and perceptions are essential for the development of legitimate regimes, by basing policy recommendations on positive experiences in the current prison setting we are naturally limited in our proposals by the conditions under which prisons *currently* operate. A prisoner cannot discuss in positive terms an experience he or she has never lived; hence, policy reforms based on Liebling’s findings are naturally limited by what already exists. As moral philosopher David Hume once famously argued, we should not deduce an ‘ought’ from an ‘is’.^40^ The fact that prisoners are satisfied with procedural rather than substantive justice does not mean that we should concentrate our reform efforts exclusively on procedural changes.

English prisons are governed through a vertical power structure that makes prisoners depend on the help and assistance of guards for virtually every aspect of their life during detention. If placed in a less regulated environment that gives prisoners more autonomy and that places more emphasis on self-determination, it is likely that the legitimacy of the institution would depend less on the actions of guards and more on the quality of prisoners’ lives while in custody. While civil and respectful treatment in a safe environment is of course a desirable goal, this should only be seen as a baseline, as a minimum starting point from which to develop reforms aimed at advancing a more purposeful use of prison time that can be said to be truly transformative in preparation for release. As argued by Watson, prisons in England ‘direct too much attention towards measuring the existence of moral values [such as humanity, justice, and respect] and only limited attention towards forging better social relations that are generative of such values in the first place’.^41^ This can be done by focusing not only on how civil interactions between staff and prisoners are, but also on how integrated prisoners are with each other and on how many opportunities prisoners are given for participation in their own governance. It can be done by complementing the current concerns with fair vertical relationships with broader concerns about the quality of horizontal relationships and about the availability of forums for democratic engagement.

The idea that legitimate regimes need to involve democratic participation naturally flows from Bottoms and Tankebe’s dialogic notion of legitimacy.^42^ As highlighted by Ian Loader and Richard Sparks in their chapter ‘Reasonable Hopes’,

in their extension of accounts of legitimacy beyond the procedural justice paradigm, [Bottoms and Tankebe] argue that legitimacy is best thought of as a matter of ongoing dialogue … this means transcending the idea that legitimacy is mainly about compliance and obedience … it also has to do with contemporary people’s expectations of voice and participation in important public questions that affect their lives … [I]f legitimacy is, for some purposes at least, an idea that suggests dialogue, then legitimate social arrangements might be the ones that make space for debates and discussions that inform actions or policies.^43^

If, as argued by these authors, ‘dialogue and deliberation are intrinsic values in democratic forms of organisation’, it follows that a model of governance based on democratic values and practices is more likely to ensure a stable version of legitimacy and compliance, even in modern prison settings.

Although both Liebling and Sparks, Bottoms and Hay have moved beyond the procedural paradigm in their accounts of legitimacy,^44^ neither of them has advanced proposals that include considerations of the potential benefits of democratic forms of participation in prison settings. A possible reason for this omission is that their work focuses primarily on prison experiences in England and Wales, where democratic forms of inmate participation are relatively rare, despite the growth of democratic experiments in recent years.^45^ To address this limitation, we should broaden our geographical lens to examine settings that are currently operating under alternative models of prison governance. As argued by O’Donnell, contemporary studies highlighting the challenges of prison life ‘tend to be limited to the Global North and in particular the anglosphere. This geographical constraint results in scholarship that is insufficiently ambitious and there is much to be gained by broadening the focus.’^46^ To imagine a more radical and ambitious reform agenda, we need to look comparatively at different experiences, institutional ideologies and modalities of power distribution to remind ourselves that another way is possible. By examining prisons that do ‘things differently’,^47^ we might discover that legitimacy and compliance can be achieved in more empowering ways than what prison research conducted in late 20th- and early 21st-century England and Wales might suggest.

The argument for democratic forms of prisoner participation is twofold. First, prisoners in the UK are denied the right to vote and are therefore hindered from influencing political decisions regarding the conditions of their confinement.^48^ This disenfranchisement is based on the view, largely shared by the political right in both Britain and the United States, that ‘inmates are not part of the *demos* and including them in institutional governance degrades both democracy and the basic conditions of confinement’.^49^ Given the repeated failures on the part of the European Court of Human Rights to pressure the UK government into lifting these laws,^50^ there is reason to believe that this disenfranchisement is there to stay, at least for the near future. Hence, if we wish to hold prison administrations accountable to the subjects they are empowered to govern, we need to give inmates a voice from inside the prison walls. Without the political power to influence decisions regarding the conditions of their captivity, prisoners might retrieve their consent and seek more disruptive ways to have their voices heard and their wishes considered. Prisons cannot be said to be truly legitimate until this democratic deficit has been addressed, no matter how fair the procedures, and how civilised staff–prisoner relations, are.

Second, history shows that democratic forms of prisoner participation and self-governance can be ‘transformational’ and ‘consciousness raising’, advancing human flourishing and social integration to an extent unknown under traditional managerial models.^51^ As Liebling argues, feelings of legitimacy and trust are essential for prisoners’ psychological well-being. In a series of studies conducted in the years following the implementation of the Moral Quality of Prison Life questionnaire, a survey used by the English Prison Service to evaluate the ‘moral performance’ of different prison settings, Liebling found that regimes that fair well on the test tend to produce better outcomes in terms of prisoners’ mental and emotional well-being.^52^

This finding shows that being treated fairly and respectfully is an *essential precondition* for prisoners’ mental health. However, it does not show that fair treatment *leads* to human flourishing. A similar inference would assume an incomplete view of psychological well-being that sees absence of mistreatment as a sufficient condition for human development and justifies the continuous relegation of prisoners to a passive role of acceptance and subordination. True human flourishing stems from active engagement and purposeful activity, not from freedom from disrespectful and degrading treatment alone. If we embrace a more holistic view of psychological well-being, we can envision a system that not only treats prisoners with humanity, but also empowers them to take important decisions concerning their lives in captivity. This can be accomplished by implementing a participatory model of prison governance that involves inmates in the daily running of the institution through active citizenship and engagement.^53^ This participatory model promotes a higher form of human flourishing than the one that can be obtained through civil treatment, ‘performative’ types of respect and top-down models of prison governance. It encourages prisoners to take an active role in their correction, allowing them to shape their own destiny in ways that are likely to enhance confidence and self-esteem, provide opportunities for meaningful socialisation and create the conditions for positive identity transformations.

## 4. Democratic Participation and Horizontal Integration: An Ethiopian Model

The case of Isir Beth,^54^ a Southern Ethiopian prison analysed by O’Donnell between 2010 and 2020, clearly illustrates the benefits of democratic models of prison governance for institutional legitimacy and prisoners’ psychological well-being. Isir Beth is located in the Southern Nations, Nationalities, and People’s Region, a rural area populated by more than 45 ethnic groups and characterised, like most of the country, by high levels of poverty and short life expectancy.^55^ The case of Isir Beth is intriguing because it demonstrates that it is possible to design, and safely maintain, a sophisticated system of inmate self-governance in contexts with limited financial resources and a reduced correctional workforce. As highlighted by O’Donnell, what differentiates this prison from most Anglo-American institutions are its low level of formal regulation and high level of horizontal integration.^56^ This means that the prison is managed through a small number of vertically imposed rules and an equally small number of prison officers, and that, partially as a result of this low level of regulation, the inmate population is highly integrated and generally cohesive.

At Isir Beth, prisoners’ high level of integration is strongly linked to the way social and economic activities are organised within the prison walls. In contrast to most facilities in the Global North, the prison is located in a large town, close to residential areas, and surrounded by the bustling everyday activities of the residential population.^57^ It can be described as a ‘gated village’, an enclosed community in which everyday life mirrors, to a large extent, life outside the prison, apart from inmates’ condition of captivity.^58^ While at night detainees are locked inside the dormitories, during the day they are free to move around the compound, engage in various income producing activities and socialise with each other without restrictions.^59^ The prison is a vibrant, active, market society, where prisoners weave cotton yarn to produce scarves and blankets, make fish nets and are busy with ‘crochet, embroidery, cooking, wood carving, and shopkeeping’.^60^ This allows them to provide for their families at home, develop a craft and deal with the boredom of imprisonment in productive ways. Visits from family members are frequent, and respectful physical contact is allowed during such visits. The place is crowded, and prisoners need each other to do business and make a living; hence, they are less likely to let temporary antagonisms degenerate into physical violence. As highlighted by O’Donnell, this type of prison organisation reflects the communitarian ideology that permeates social relations in Ethiopia: ‘Ethiopians are accustomed to community organisation. Ethiopian society is more collectivistic than individualistic, so it makes sense that this tendency would be reflected, perhaps even magnified, in the prison.’

### A. Governance and Safety

At Isir Beth, there is a clear division of labour between correctional officers and prisoners in the daily running of the institution. Correctional officers are in charge of supervising the perimeter of the prison, managing and searching the flow of visitors, and locking and unlocking the dormitories for the night curfew.^61^ Prisoners, on the other hand, are in charge of managing every other aspect of prison life (through a range of elected committees), including preventing the building of hostile relationships within the compound and taking disciplinary actions.^62^ For this role, they have devised a system of dormitory keepers, who can be identified by the purple hat that they wear while on duty.^63^ Despite their authoritative position, the keepers enjoy a high level of legitimacy amongst prisoners both because they are elected by the dormitories and because, being inmates themselves, they are more sensitive to the needs and dynamics of the prison population.^64^

Relations between staff and prisoners are ‘distant, but cordial’; they lead relatively independent lives, with officers being more focused on preventing escapes than on controlling prisoners’ daily activities and associations.^65^ This lack of regulation and control, and the system of self-governance that stems from it, is not a deliberate choice taken by the prison authorities based on an anarchic or democratic socialist ideology. Rather, it is a direct consequence of the lack of resources that pervades the system, which forces authorities to prioritise general incapacitation over daily micromanagement.^66^ In this context, prisoners fill the power void left by the government by devising a system of community living based on transparent rules, democratic participation and daily co-operation. This system of order maintenance has been highly successful, as reflected both in the low levels of violence registered within the compound and in the feelings of safety reported by the prison population.^67^

### B. Legitimacy and Compliance

At Isir Beth, legitimacy and compliance stem from the implementation of a sophisticated system of democratic participation that sees all parties involved in the creation of rules for good community living. In 2015, the prisoners devised a written code of conduct to substitute subjective decision making with fair, transparent and predictable outcomes.^68^ The code was officially signed and approved by 90% of the prison population and is regularly revised by prisoners in ‘refresher sessions’ aimed at ensuring that the rules are still relevant and that there is ‘ongoing consensus about the dictates of everyday life’.^69^ By giving prisoners a forum to voice any issues stemming from the daily implementation of the rules, this system ensures (i) that the code maintains a high level of legitimacy amongst the prison population and (ii) that the keepers who are in charge of ensuring compliance with the rules can do so without too much resistance.^70^ The legitimacy and compliance that ensues can be said to be truly normative, because it is based on the shared values and beliefs that emerged at the time of drafting of the code as well as during the periodical refresher sessions. As argued by O’Donnell, legitimacy at Isir Beth is strong and stable because:

written constitutions … [are] not imposed on prisoners. Rather, they originat[e] from them, [are] endorsed by them, and [become] moral imperatives that they subscrib[e] to and [are] obligated to promote. Everyone sacrifice[s] a measure of personal autonomy in the interests of collective security and what [is] perceived as the public good. This process unfold[s] against a background of carceral regulation that [is] light of touch and largely irrelevant. Prisoners identif[y] a need and then initiat[e] a deliberative process to find a mutually satisfactory way of meeting it; governance [is] actively and far-sightedly brought about.^71^

At Isir Beth, many prisoners sleep in overcrowded dormitories, eat low-quality food and have insufficient water to drink and maintain personal hygiene.^72^ Nevertheless, prison disorders and riots are virtually non-existent, assaults and batteries are rare, and prisoners feel generally safe.^73^ The reasons for this can be found both in the high level of horizontal integration found amongst the prison population as a result of their socio-economic organisation and in the high levels of legitimacy enjoyed by the institution as a result of inmates’ involvement in rule creation and implementation. This dual version of legitimacy (*horizontal* and *vertical*)^74^ creates the conditions for more stable forms of compliance than the one that can be obtained in contexts where only vertical legitimacy is present, as happens in many prisons in Britain and the United States.

## 5. Democratic Participation and Prisoner Reintegration: A Brazilian Model

Another example of inmate participation that has led to reductions in inmate-to-inmate violence can be found in the alternative prison model developed by the Association for the Protection and Assistance of Convicted Persons (Associação de Proteção e Assistência a Condenados, APAC) in Brazil. The APAC is a Christian, non-profit non-governmental organisation that aims to provide better conditions for detainees through institutions inspired by the values of ‘love, forgiveness, and transformation’ rather than by the principle of retribution.^75^ The central aim of APAC ‘prisons’ is the social reintegration of inmates (the APAC calls the model an ‘educational system of social reintegration’), to be obtained through a rehabilitation programme based on study, vocational training and religious faith. As explained by Valdeci Ferreira, Executive Director of the Brazilian Fraternity of Assistance to the Convicted (an organisation that co-ordinates all the APAC centres in Brazil), the ‘APAC was born to recover the inmate, protect society, assist victims and promote restorative justice. To achieve these objectives, we apply our own criminal therapy, which we call the APAC method.’^76^ To participate in the programme, prisoners need to fill in a formal application in which they commit to the rules of the APAC and to its rehabilitation method.^77^ If accepted in the programme, they will be transferred from the traditional prison setting to the APAC unit. Once in the APAC unit, they are expected to spend 15 hours a day (from 7:00 am to 10:00 pm) outside of their cells, working, studying and engaging in other activities.^78^

The first APAC prison was founded in 1972 in São José dos Campos in São Paulo, in response to a series of inmate protests that prompted administrators to seek the help of volunteers who knew the prisoners involved in the riots. The APAC now holds units in various locations, both in Brazil and across the world, supporting approximately 48,501 *recuperandõs* (the name given to inmates participating in the programme) since the year of its foundation. The rapid spreading of the APAC system can be explained by the low recidivism rates registered amongst released detainees,^79^ the cost-effectiveness of the model and the low levels of violence registered within the units compared to traditional Brazilian prisons. While these low levels of violence can be partially explained by the conditions of participation in the programme, which require inmates to commit to the APAC method and to follow the rules of the units, they are not related either to selected inmates’ criminal records or to the number of infractions they committed while in traditional prison detention.^80^ Indeed, many *recuperandõs* had been convicted for very serious offences, including homicide and rape, and had committed serious disciplinary infractions while in traditional prison settings.

### A. Communication, Awareness Generation and Compliance

The key objective of the APAC model is social reintegration, to be achieved through education, professionalisation and socialisation. The activities of each APAC unit are conducted in centres for social reintegration (*centro de reintegração social*, CRS), which are described as ‘peaceful places with relaxed and aesthetically pleasing atmospheres’ designed with reintegration activities in mind.^81^ Inside the CRS, *recuperandõs* wear normal clothing, are addressed by their own name and are able to interact and create social bonds, both with each other and with the APAC volunteers who run the activities of the centres.^82^ Social cohesion and prisoners’ integration are core objectives of the educational programmes promoted in CRSs, which are peppered with informal activities aimed at facilitating vertical and horizontal communication within the units. As social educator Sergio Grossi explains in his analysis of CRSs:

Coexistence in the units is an educational issue with a variety of open channels of communication with the administration, ranging from dormitory meetings to collective meetings. The daily routine of interaction and coexistence among prisoners, who cannot practice any form of violence, is also described as an ‘awareness generator’.^83^

In this model, inmates’ involvement in various forums of deliberation and discussion is primarily seen as a formative experience that can lead to consciousness raising and to the building of more tolerant and respectful relationships. However, the absence of riots and rebellions in the units suggest that, in addition to contributing to prisoners’ education, these daily discussions enhance the legitimacy of the system, encouraging prisoners to comply with the rules of the CRSs and refrain from disruptive behaviours.

### B. Governance without Force

The model of governance used in CRSs differs dramatically from the one found in traditional prisons, both in Brazil and in the Global North.^84^ Here, guards are not allowed to carry arms, and security is maintained without the use of force. This is possible thanks to a ‘security policy based on personal relationships between operators and prisoners’ that places a premium on human dignity and that draws on clear and well-established rules of community living.^85^ In contrast to what happens in most prisons in the Global North, in these institutions trust between staff and inmates is built both through fair relationships and through *recuperandõs*’ involvement in the daily co-running of the facilities.^86^ In CRSs, *recuperandõs* are not reduced to passive beings without identity or agency. Instead, they are acknowledged in their individuality and involved in the management of every aspect of the CRSs, including order maintenance and safety. As Grossi highlights, in APAC units

prisoners participate in the management of the centre, creating a potentially healthier environment for reintegration … prisoners also participate in the administration of the prison, which continues to be a specificity of the model—the rigid division (typically found in traditional prisons) between the guards and the people deprived of their freedom is eliminated. In APAC units, the role of maintaining discipline and preventing escape is also attributed to everyone inside the units generating a new organisation.^87^

As happened in Isir Beth, in CRSs power and authority are not defined by vertical power relations between officers and prisoners. Instead, they are shaped by a sense of shared responsibility for the management of a collective space, in which all actors involved work together to induce compliance with the rules of the unit, prevent violent conflicts and ensure the smooth running of the centre’s daily activities.^88^

To engage prisoners in order maintenance, CRSs use a number of strategies. One such strategy involves the implementation of a code of honour that praises and rewards *recuperandõs* who report infractions. This is done by depicting informants as committed and trustworthy and by giving them positions of responsibility and leadership within the unit. By rewarding inmates who report rule breaking, this code inverts the hierarchy of authority found amongst inmates in traditional prisons, where whistleblowers are ostracised and inmates following the code of silence are respected.^89^ While this strategy might lead to opportunistic behaviours on the part of prisoners who act as whistleblowers to climb the units’ hierarchical ladders, research has shown that this change of culture has led to positive identity transformations amongst the *recuperandõs* committed to the programme. This suggests that behaviours that are initially prompted by utilitarian considerations can eventually be internalised, leading to the embracement of values that align more strongly with those of the institution and laying the foundations for a truly normative commitment towards compliance.

## 6. Conclusion

The idea of democratic participation and inmate self-governance is not a utopian and naive project. Rather, it falls within the scope of what philosophical pragmatist Roberto Unger calls the ‘adjacent possible’, ‘the link between insight into what exists and imagination of what might exist at the next steps’.^90^ As the case studies analysed in this review article show, democratic experiments have proven to be successful in institutions with different characteristics and placed in different geographical contexts, sometimes under conditions of very limited financial resources. What is needed for the implementation of democratic forms of prison participation is not an outpouring of state funding, but the political will to rethink the current model of governance and the courage to advocate more radical reforms that trust prisoners to be able to take an active role in their own correction. In her studies, Liebling consistently found that prisoners were ‘enthusiastic participants in moral conversation’.^91^ What makes us think that they cannot be equally enthusiastic—and, I would add, competent—participants in political self-governance?

As mentioned in section 1, the past few years have seen significant turmoil in English prisons, with officials reporting chronic overcrowding, unsanitary conditions and record levels of violence, suicide and self-harm in the majority of the facilities. At the same time, a growing number of institutions are experimenting with new instruments for inmate involvement, offering people in prison opportunities to become active citizens through participation in advisory boards and volunteering.^92^ As argued by Schmidt, ‘this is a historical moment of both crisis and possibility that presents the opportunity to take up the challenge of reimagining civic engagement and social transformation with some political creativity toward reconfiguring institutional order and change’.^93^

In their work on legitimacy in prisons, Sparks, Bottoms and Hay and Liebling emphasise how relationships based on fair and respectful treatment can help build trust in criminal justice institutions and enhance psychological well-being, two important features in contexts typically characterised by loss and suffering, such as the English prison system. This argument largely confirms the procedural paradigm developed by Tyler, who, in his influential book *Why People Obey the Law*, found that the legitimacy of criminal justice institutions depends more on whether citizens perceive legal officials to act fairly than on whether they perceive officials’ decisions to be fair.^94^ However, Sparks, Bottoms and Hay and Liebling expand on Tyler’s work, acknowledging, for example, ‘that in custodial institutions, perceived outcome fairness as well as procedural fairness can be of great importance to the achievement of staff legitimacy in the eyes of prisoners’.^95^ This finding shows that, despite its powerful appeal, the procedural justice paradigm is ‘limited in scope’, and criminal justice research would benefit from theoretical and empirical work that draws a more complete picture of the roots of legitimacy and social order.^96^

Legal scholar David J Smith has importantly stressed that to provide a fuller account of legitimacy we need to broaden our focus beyond the US (and UK) context, to learn from the experiences of societies with radically different ideologies and political arrangements.^97^ The procedural justice paradigm, he argues,

is based on research into the views and self-reported experiences of individuals in one society at one point in history [the United States in the 1990s], although, importantly, it is found to apply equally to men and women, to different age and ethnic groups, and to different social classes within that society. There is strong temptation to move from that densely populated but restricted evidence base to the general conclusion that fair procedures are always and everywhere the main foundation of legitimacy.^98^

Rather than drawing overly broad inferences from Tyler’s findings, Smith argues, scholars should acknowledge this limitation and explore how legitimacy is established and maintained in ‘contemporary societies with political systems and social and economic conditions that stand in marked contrast to those existing in the United States’.^99^ As the case studies analysed in this review article show, a similar broadening might bring into focus different values and priorities that question the legitimacy of procedurally fair, but substantially unjust, social institutions, while challenging the traditional power imbalance existing between legal authorities and subordinated subjects in Anglo-American penal contexts.

## Footnotes

1
*Crime, Justice, and Social Order*, ch 12. See also Anthony Bottoms, ‘Criminology and “Positive Morality”’ in ibid ch 3.

2 Ian Loader and Richard Sparks, ‘Reasonable Hopes: Social Theory, Critique, and Reconstruction in Contemporary Criminology’ in ibid ch 6; Justice Tankebe, ‘Rightful Authority: Exploring the Structure of Police Self-Legitimacy’ in ibid ch 14.

3 Richard Sparks, Anthony Bottoms and Will Hay, *Prisons and the Problem of Order* (OUP 1996).

4 For the first procedural account of legitimacy in criminal justice contexts, see Tom R Tyler, *Why People Obey the Law* (Yale UP 1990).

5 Pamela Duncan and others, ‘The State of Prisons in England and Wales in Numbers’ *The Guardian* (London, 26 September 2023) <www.theguardian.com/society/2023/sep/26/the-state-of-prisons-in-england-and-wales-in-numbers#:~:text=Safety%20in%20prisons%20has%20deteriorated,thirds%20between%202020%20and%202022> accessed 5 July 2024.

6 Rachel Sylvester, ‘Britain’s Prisons Are a Tinderbox—One Spark and It May Kick Off’ *The Times* (London, 23 April 2024) <www.thetimes.com/uk/law/article/crime-justice-commission-britain-prisons-tinderbox-2sddlcs92> accessed 5 July 2024.

7 ibid.

8 ibid.

9 Liebling (n 1) 278; Gabrielle Watson, *Respect and Criminal Justice* (OUP 2020) 104–5.

10 Watson (n 9) 104.

11 Liebling (n 1) 278.

12 ibid.

13 ibid 279.

14 ibid.

15 ibid 280.

16 ibid 281. For other studies showing that prisoners’ perceptions of fairness depend more on the perceived fairness of staff than on the perceived fairness of the regime, see Suhail Ahmad, ‘Fairness in Prisons’ (PhD thesis, University of Cambridge 1996).

17 Liebling (n 1) 283.

18 ibid 285.

19 ibid.

20 ibid 285–6 (emphasis added).

21 ibid 290.

22 ibid 300. This finding was again confirmed in Alison Liebling and Helen Arnold, *Prisons and Their Moral Performance: A Study of Values, Quality, and Prison* Life (OUP 2004), an appreciative inquiry conducted in five English prisons in the early 2000s. In her chapter contribution on women community centres, Loraine Gelsthorpe found that women’s feelings of legitimacy and compliance were similarly sensitive to respectful and caring treatment by staff. See Loraine Gelsthorpe, ‘Keeping a Human Perspective: Some Lessons from Women Community Justice’ in *Crime, Justice, and Social Order* 144–5, 149–50.

23 Tom Tyler, ‘Trust and Legitimacy: Policing in the USA and Europe’ (2011) 8 European Journal of Criminology 254, 257.

24 Anthony Bottoms and Justice Tankebe, ‘“A Voice Within”: Power-holders Perspectives on Authority and Legitimacy’ in Alison Liebling and Justice Tankebe (eds), *Legitimacy and Criminal Justice: An International Exploration* (OUP 2013) 62 (emphasis added).

25 Duncan and others (n 5).

26 Ian O’Donnell, *Prison Life: Pain, Resistance, and Purpose* (New York UP 2023) 230. This view aligns with the notion of legitimacy developed by David Beetham in *The Legitimation of Power*. According to Beetham, normative compliance is a necessary precondition for truly legitimate social institutions. If compliance is obtained through other means, the institution is more likely to lose its legitimacy in the eyes of the audience when it fails to fulfil expectations, making its perceived authority less stable and durable. See David Beetham, *The Legitimation of Power* (2nd edn, Bloomsbury Academic 2013).

27 O’Donnell (n 26) 230.

28 Richard Sparks and Anthony Bottoms, ‘Legitimacy and Order in Prisons’ (1995) 1 British Journal of Sociology 46, 58–9 (emphasis added).

29 See eg Tyler, *Why People Obey the Law* (n 4).

30 Ronald L Cohen, ‘Fabrications of Justice’ (1989) 3 Social Justice Research 31, 38.

31 Watson (n 9) 130–1.

32 ibid.

33 Susie Hulley, Alison Liebling and Ben Crewe, ‘Respect in Prisons: Prisoners’ Experiences of Respect in Public and Private Sector Prisons’ (2012) 12 Criminology & Criminal Justice 3, 5 (emphasis added).

34 Richard Sparks and Anthony Bottoms, ‘Legitimacy and Imprisonment Revisited: Some Notes on the Problem of Order Ten Years After’ in James Byrne, Don Hummer and Faye Taxman (eds), *The Culture of Prison Violence* (Pearson Education 2008) 102. For a discussion of the concept of ‘dull compulsion’, see also Eamonn Carrabine, *Power, Discourse and Resistance: A Genealogy of the Strangeways Prison Riot* (Ashgate 2004).

35 Sparks and Bottoms, ‘Legitimacy and Imprisonment Revisited’ (n 34) 102 (emphasis added).

36 Bethany E Schmidt, ‘Democratising Democracy: Reimagining Prisoners as Active Citizens through Participatory Governance’ (PhD thesis, University of Cambridge 2020).

37 A question raised by Sparks, Bottoms and Hay in *Prisons and the Problem of Order* (n 3). See Schmidt (n 36) 1.

38 Schmidt (n 36) 3. See also Amy E Lerman and Vesla M Weaver, ‘A Tradeoff between Safety and Democracy? An Empirical Investigation of Prison Violence and Inmate Self-governance’ in Albert W Dzur, Ian Loader and Richard Sparks (eds), *Democratic Theory and Mass Incarceration* (OUP 2016).

39 See Liebling and Arnold (n 22).

40 David Hume, *A Treatise of Human Nature* (first published 1739, OUP 2014) bk III, pt I, para i; Max Black, ‘The Gap between “Is” and “Should”’ (1964) 73 Philosophical Review 165, 165.

41 Watson (n 9) 119.

42 Anthony Bottoms and Justice Tankebe, ‘Beyond Procedural Justice: A Dialogic Approach to Legitimacy in Criminal Justice’ (2012) 102 J Crim L & Criminology 119, 129; Tankebe (n 2) 337–40.

43 Loader and Sparks, ‘Reasonable Hopes’ (n 2) 116–17.

44 As discussed in their work, these scholars have expanded the procedural approach to prison legitimacy by highlighting the importance of humane treatment, the quality of staff–prisoner relationships, institutional alignment with prevailing values and sensibilities, and inmates’ psychological well-being, for example. See Sparks, Bottoms and Hay (n 3) 327, 329; Sparks and Bottoms, ‘Legitimacy and Order in Prisons’ (n 28) 58–9; Liebling and Arnold (n 22) 475.

45 See eg Schmidt (n 36).

46 O’Donnell (n 26) 1.

47 Loader and Sparks, ‘Reasonable Hopes’ (n 2) 101.

48 House of Commons Library, *Prisoners’ Voting Rights* (Research Briefing No 7461, 2023).

49 Christopher D Berk, ‘On Prison Democracy: The Politics of Participation in a Maximum Security Prison’ (2018) 44 Critical Inquiry 275, 280. See also Christopher D Berk, *Democracy in Captivity: Prisoners, Patients, and the Limits of Self-Government* (UCP 2023) 79.

50 Ergul Celiksoy, ‘Execution of the Judgments of the European Court of Human Rights in Prisoners’ Right to Vote Cases’ (2020) 20 Human Rights Law Review 555, 569–75.

51 Schmidt (n 36) 85, 128–9. Carolyn Eggleston and Thom Gehring, ‘Democracy in Prison and Prison Education’ (2000) 51(3) Journal of Correctional Education 306, 308.

52 Liebling (n 1) 290–2.

53 As Christopher Berk argues in his analysis of the Walpole prison democratic experiment, ‘inmates self-governance is a form of applied civic education, where one develops a capacity for complex forms of participation by engaging in more basic participatory acts’. Berk, ‘On Prison Democracy’ (n 49) 296.

54 Isir Beth, which means ‘prison’ in Amharic, is a fictitious name invented by O’Donnell to maintain the anonymity of the institution. O’Donnell (n 26) 127.

55 ibid 127–8. In 2019, the per capita gross national income in Ethiopia was US$850 and the average life expectancy was 67 years. ibid.

56 ibid 3–4.

57 ibid 128.

58 ibid.

59 ibid 131, 165–6.

60 ibid 136.

61 ibid 155, 162.

62 ibid 151–4.

63 ibid 154.

64 ibid 155.

65 ibid 4, 162.

66 ibid 161.

67 ibid 157–8.

68 ibid 142.

69 ibid 145.

70 ibid 145–6.

71 ibid 9.

72 ibid 132–3.

73 ibid 157–8.

74 ibid 17–8.

75 Prison Fellowship International, ‘APAC Is Changing Lives in Brazil’ <https://pfi.org/resources/stories-of-hope/apac-is-changing-lives-in-brazil/> accessed 5 July 2024.

76 Justice Trends, ‘APAC: Prisons without Guards, Police, Weapons, Violence, Corruption, Drugs and Discrimination’ (*Justice Trends*, 21 July 2020) <https://justice-trends.press/the-apac-revolution-prisons-without-guards-without-police-without-weapons-without-violence-without-corruption-without-drugs-without-discrimination/#:~:text=Mario%20Ottoboni.,we%20call%20the%20APAC%20method> accessed 5 July 2024.

77 Sergio Grossi, ‘Rethinking Social Reintegration and Prison: A Critical Analysis of an Educational Proposal for an Alternative Model in Brazil’ (2021) 7 Journal of Prison Education and Reentry 183, 186.

78 ibid.

79 According to Grossi, APAC units’ recidivism rates range from 8 to 20%, whereas Brazil’s national prison system’s rates are around 70%. ibid 184.

80 ibid 185–6.

81 ibid 185.

82 ibid 186.

83 ibid.

84 See Luiz Dal Santo, ‘Brazilian Prisons in Times of Mass Incarceration: Ambivalent Transformations’ (2002) 61 The Howard Journal of Crime and Justice 502, 502–18.

85 Grossi (n 77) 185.

86 ibid 188.

87 ibid.

88 ibid.

89 ibid 191.

90 Roberto Unger, ‘What Is Wrong with the Social Sciences Today?’ (*Social Science Bites*, 9 January 2014) <www.socialsciencespace.com/2014/01/roberto-mangabeira-unger-what-is-wrong-with-the-social-sciences> accessed 6 July 2024; Loader and Sparks, ‘Reasonable Hopes’ (n 2) 121. See also Roberto Unger, *The Self Awakened: Pragmatism Unbound* (Harvard UP 2007) 86.

91 Liebling (n 1) 292.

92 Schmidt (n 36) 5 (citations omitted).

93 ibid.

94 Tyler, *Why People Obey the Law* (n 4).

95 Bottoms and Tankebe, ‘Beyond Procedural Justice’ (n 42) 123. See also Sparks, Bottoms and Hay (n 3) 303–11and Liebling and Arnold (n 22) 475.

96 Bottoms and Tankebe, ‘Beyond Procedural Justice’ (n 42) 123. For a compelling critique of the procedural justice paradigm in policing research, see Dorian Schaap and Elsa Saarikkomäki, ‘Rethinking Police Procedural Justice’ (2022) 26 Theoretical Criminology 416, 416–33.

97 David J Smith, ‘The Foundations of Legitimacy’ in Tom R Tyler (ed), *Legitimacy and Criminal Justice: An International Perspective* (Russell Sage Foundation 2007) 31–6.

98 ibid 31.

99 ibid 33.

